# Novel Design Strategies to Enhance the Efficiency of Proteolysis Targeting Chimeras

**DOI:** 10.1021/acsptsci.2c00089

**Published:** 2022-08-22

**Authors:** Chunlong Zhao, Frank J. Dekker

**Affiliations:** Department of Chemical and Pharmaceutical Biology, Groningen Research Institute of Pharmacy (GRIP), University of Groningen, Antonius Deusinglaan 1, 9713AV Groningen, The Netherlands

**Keywords:** targeted protein degradation (TPD), proteolysis targeting chimera (PROTAC), new drug modality, photochemical control, hypoxia activation

## Abstract

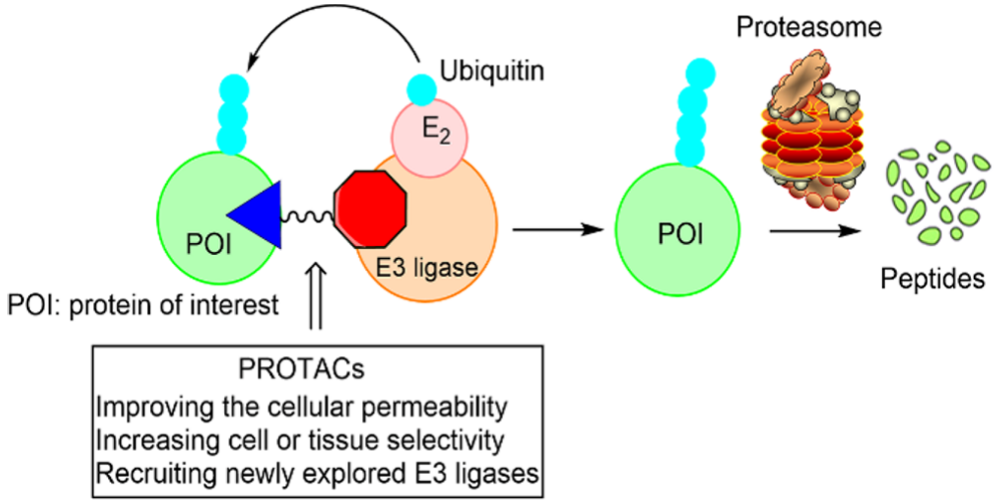

Despite the success of drug discovery over the past decades, many potential drug targets still remain intractable for small molecule modulation. The development of proteolysis targeting chimeras (PROTACs) that trigger degradation of the target proteins provides a conceptually novel approach to address drug targets that remained previously elusive. Currently, the main challenge of PROTAC development is the identification of efficient, tissue- and cell-selective PROTAC molecules with good drug-likeness and favorable safety profiles. This review focuses on strategies to enhance the effectiveness and selectivity of PROTACs. We provide a comprehensive summary of recently reported PROTAC design strategies and discuss the advantages and disadvantages of these strategies. Future perspectives for PROTAC design will also be discussed.

## 1 Introduction

In the past several decades, traditional occupancy-driven small-molecule compounds, which inhibit target proteins function by occupying their active or allosteric sites, have achieved great success in drug discovery.^1−3^ However, occupancy-driven drug discovery is not feasible for all drug targets. This is demonstrated by the estimate that nearly 400 human proteins are disease-related, whereas they lack a binding pocket for small molecule drug discovery, which renders them undruggable.^3−6^ Another issue is therapy resistance that can emerge upon long-term treatment with inhibitors, especially in the kinase inhibitor field.^2,7^ In addition, some proteins have scaffolding functions which cannot be targeted by traditional inhibitors.^8−13^ Therefore, there is an urgent need for novel strategies to address these limitations.

**Figure 1:**
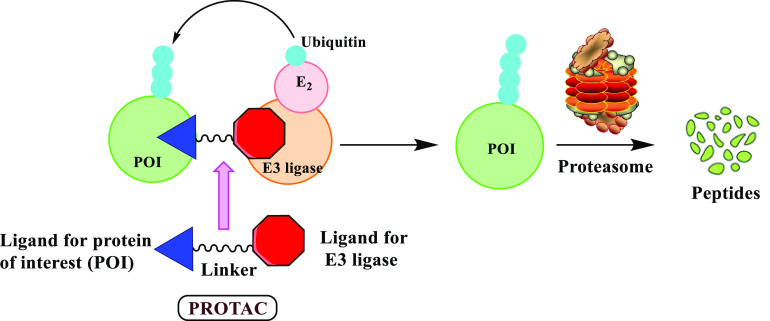
Schematic representation of proteolysis targeting chimeras (PROTACs). The PROTAC molecule is a heterobifunctional compound containing an E3 ligase recruiter (in red) and a ligand for the protein of interest (POI) (in blue). PROTACs induce the proximity of the E3 ligase and the POI to trigger ubiquitination (in light blue) and subsequent proteasomal degradation (in light green).

Targeted protein degradation (TPD) by proteolysis targeting chimeras (PROTACs) offers a new paradigm in drug discovery. A PROTAC molecule is a heterobifunctional compound consisting of a ligand for the protein of interest (POI), a ligand for ubiquitin E3 ligase, and a linker (Figure 1).^3,14^ This heterobifunctional character enables simultaneous binding to the POI and a ubiquitin E3 ligase to form a ternary complex, which activates the ubiquitin-proteasome system (UPS) to degrade the POI (Figure 1).^3,14,15^ The action of PROTACs is event-driven, which enables PROTACs to act catalytically as one PROTAC molecule could trigger degradations of multiple molecules of the POI. Both the ability to trigger protein degradation and the event-driven mode of action render PROTACs a unique therapeutic potential in comparison to classical occupancy-driven therapeutics.^16,17^ For example, PROTACs enable therapeutic exploitation of proteins that were previously considered undruggable.^5,18−22^ Apart from interfering with the ligand interaction site, PROTACs trigger protein degradation, which also enables inhibition of protein scaffolding functions or protein–protein interactions that could previously not be targeted.^10−12,21,23^ Moreover, PROTACs may hold potential to overcome drug resistance.^21,24−28^ For example, it has been shown that several PROTACs triggered the degradation of Bruton’s tyrosine kinase and its mutants, which demonstrates the potential to treat mutation-induced ibrutinib-resistant diseases.^27,28^

**Figure 2:**
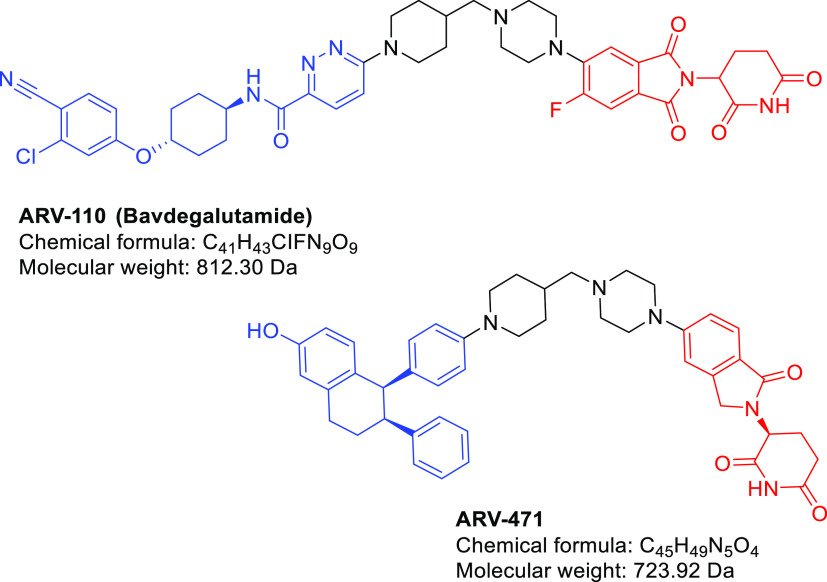
Chemical structures of ARV-110 (Bavdegalutamide) and ARV-471. ARV-110 is an androgen receptor (AR) degrader, while ARV-471 is an estrogen receptor (ER) degrader. The E3 ligase ligands are shown in red, the POI ligands are shown in blue, and the linkers are shown in black.

The clinical importance of the PROTAC strategy has been demonstrated by the entry of over ten PROTAC-based molecules in clinical trials.^29^ The chemical structures of the PROTACs ARV-110 (NCT03888612) and ARV-471 (NCT04072952) are shown in Figure 2. These PROTACs target the androgen receptor (AR) or estrogen receptor (ER), respectively, and have now moved on to phase II trials, which demonstrates both safety and efficacy for these PROTACs in patients.^29,30^ Despite the promising progress of PROTACs, some disadvantages may limit the success of PROTACs in the clinic. The unique chemical composition makes PROTAC molecules go beyond Lipinsk’s rule of five and may negatively affect their pharmaceutical effects.^19,31−33^ For example, PROTACs have higher molecular weight (MW) compared to traditional small-molecule inhibitors, which may impose a pharmacokinetic hurdle to their cell permeability.^33,34^ Interestingly, recent studies demonstrate that chameleonic properties play a role in enabling cell-permeability of PROTACs. For example, Kihlberg’s group found that a PROTAC with a PEG-linker populated conformations that matched the environments. The PROTAC adopted elongated and polar conformations in aqueous solvents that mimic the extra- and intracellular compartments. However, the PROTAC adopted conformations with a smaller polar surface area in apolar solvents (chloroform) that mimic the cell membrane interior.^35^ This finding suggests that PROTACs flexibility may enable chameleonic behavior in which the PROTAC surface adopts to the solvent in order to enable good solubility in both polar and apolar compartments, thus enabling cellular permeability.^35,36^ Another disadvantage of PROTACs is the potential toxicity which is caused by either on-target protein degradation in normal tissues or off-target protein degradation.^6,37−39^ Compared to traditional small molecules, PROTACs normally have more complexities with regard to drug metabolism and pharmacokinetics (DMPK) and safety evaluation.^33,40^ PROTACs have shown “hook effects” at high concentrations in which a target protein-PROTAC or E3 ligase-PROTAC binary complex can be formed competitively, resulting in decreased efficacy.^19,37,41^ Additionally, the metabolites of PROTACs, especially the metabolites in which the linker is cleaved, may competitively bind to the POI or the E3 ligase, which can antagonize degradation of the POI, thereby reducing the efficacy of the parental PROTAC.^40^ Therefore, establishment of novel approaches to characterize the pharmacokinetics and metabolite profiling is needed. Thus far, a limited number of E3 ligases (e.g., CRBN, VHL, MDM2, IAP) are used for PROTAC development.^36,42,43^ However, drug resistance for PROTACs containing CRBN and VHL E3 ligase recruiters in tumors has already emerged.^44^ Besides, these E3 ligases are generally considered to be ubiquitously expressed in humans, which show limited selectivity of PROTACs in cancer cells over normal cells. Moreover, some CRBN-based PROTACs could also lead to degradation of CRBN neo-substrates, leading to off-target toxicity.^38,39,45^ As a result, there is also hope for exploration of other E3 ligases, especially the tissue or disease-specific of E3 ligases.

Because PROTACs proved to have limited selectivity and cell-permeability,^19,37^ novel strategies have been developed to increase the effectiveness and selectivity of the PROTAC technology. These new PROTAC design strategies can be classified into three main groups: strategies for improving the cellular permeability, strategies for increasing cell or tissue selectivity, and strategies for recruiting newly exploited E3 ligases. These novel strategies enable the improvement of the effectiveness and selectivity, although further improvement with respect to DMPK study and safety evaluation is needed for the clinical success. On the basis of these new strategies, future perspective for PROTAC design will also be discussed.

## 2 PROTACs for Improving Cellular Permeability

**Figure 3:**
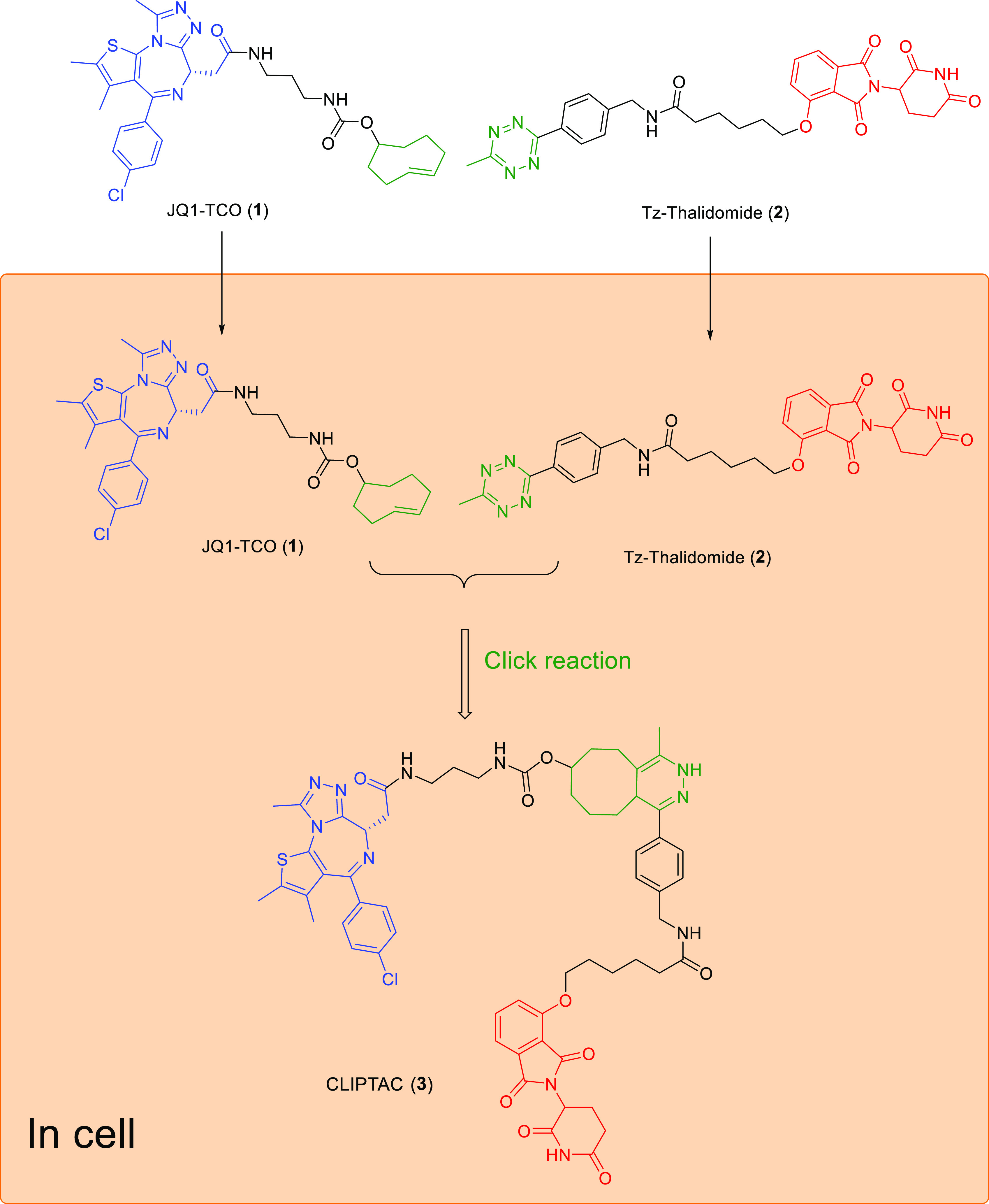
Schematic representation of the mode of action of click-formed PROTACs (CLIPTACs). Cells are treated by the TCO-tagged ligand for a target protein (in this example, BRD4), followed by a tetrazine-tagged E3 ligase recruiter (in this example, thalidomide). Click reaction happens intracellularly by the combination of two smaller precursors, generating the CLIPTAC degrader.

One major hurdle for PROTACs is their lack of compliance with Lipinsk’s rule of five because of their unique chemical composition.^19,31,33^ The MW of PROTACs generally is over 700 Da and also the rules on lipophilicity, hydrogen bond donors (HBD), acceptors (HBA), or polar surface area (PSA) are not kept.^31,33^ These unfavorable physical-chemical properties of PROTACs can lead to poor solubility and cell-permeability. Nevertheless, we note that the Lipinski’s rule of five is an empirical rule and that compliance with this rule is meant to give an estimate of the probability for a given compound to be orally bioavailable. In order to improve the cellular permeability of PROTACs, a strategy called in-cell click-formed proteolysis targeting chimaeras (CLIPTACs) was developed by Lebraud et al.^46^ The CLIPTACs strategy allows for a bio-orthogonal click reaction for the *in vivo* synthesis of PROTACs from two smaller fragments that are presumed to be more cell permeable. As shown in Figure 3, cells were treated with *trans*-cyclo-octene (TCO)-tagged ligand for BRD4 (JQ1-TCO, **1**), followed by a tetrazine-tagged E3 ligase recruiter (Tz-Thalidomide, **2**). An intracellular click reaction generated the CLIPTAC degrader (**3**), leading to degradation of BRD4. To confirm that the observed BRD4 degradation was mediated by in-cell click formation of CLIPTAC **3**, CLIPTAC **3** was synthesized by reaction of the two click precursors prior to addition to cells.^46^ Interestingly, no obvious BRD4 degradation was observed if cells were treated with preclicked CLIPTAC **3**. The results indicated the poor cell-permeability of the preclicked CLIPTAC **3** and also confirmed that the observed BRD4 degradation was ascribed to CLIPTAC **3** generated by its two precursor molecules subsequent to their entry into cells.^46^

## 3 PROTACs for Increasing Cell or Tissue Selectivity

The success of PROTACs to achieve TPD calls for therapeutic applications of this novel modality. However, their potential use is limited by both potential on-target and off-target toxicity.^37,47^ Because PROTACs are event-driven modalities, their potential toxicities are expected to be less severe in comparison to traditional on-target inhibitors. Moreover, the catalytic character of PROTACs enables the use of lower doses. Nevertheless, PROTACs might exert a serious level of on-target toxicity by degradation of the POI in areas remote from the diseased area. For example, the lack of selectivity for PROTAC-mediated proteolysis in cancer tissue versus normal tissue raises concerns about toxicity. In order to reduce the potential toxicity, several targeted PROTACs have been developed to improve cell or tissue selectivity. Promising examples of targeted PROTACs include photochemically controllable PROTACs (PHOTACs), hypoxia-activated PROTACs, folate-caged PROTACs, antibody-PROTAC conjugates (Ab-PROTACs), aptamer-PROTAC conjugates (APCs), and BCL-X_L_ PROTACs.

### 3.1 Photochemically Controllable PROTACs (PHOTACs)

The use of photochemical modulation of PROTAC activity enables spatiotemporal control of PROTAC-mediated protein degradation, which has potential to avoid side effects.^48−50^ Photocaged and photoswitchable PROTACs have been extensively investigated as reviewed.^47,48,51^ Here, we demonstrate the potential of this approach with promising examples of photocaged and photoswitchable PROTACs.

#### 3.1.1 Photocaged PROTACs

The photocaged PROTACs can be designed by caging the POI ligand or the E3 ligase ligand, thus leading to an inactive degrader. Upon light irradiation, the caging group will be removed to release the active PROTACs, which is followed by subsequent target protein degradation.^49,50,52^ In 2019, based on reported BRD4 degrader dBET1,^53^ Xue et al. designed a photocaged BRD4 degrader (Table 1, entry 4) via the installation of the 4,5-dimethoxy-2-nitrobenzyl group to the amide nitrogen of the JQ1 moiety.^52^ However, upon irradiation (365 nm), PROTAC **4** generated about 50% of the desired product dBET1. The further biological evaluations showed that the binding affinity of PROTAC **4** to BRD4 was significantly decreased. Upon irradiation with UV light at 365 nm for 3 min, 0.3 μΜ PROTAC **4** could significantly reduce the BRD4 level, comparable to the effect of 0.1 μΜ dBET1.^52^ In addition, upon irradiation, PROTAC **4** exhibited potent antiproliferative activity against Burkitt’s lymphoma cells (GI_50_ = 0.4 μΜ), similar to that of dBET1(GI_50_ = 0.34 μΜ).^52^ Unlike the Xue et al. research, Naro et al. used a 6-nitropiperonyloxymethyl group to cage the thalidomide moiety of BRD4 PROTAC.^50^ The generated photocaged BRD4 degrader **5** (Table 1, **entry 5**) could also release the parental compound and achieve BRD4 degradation in HEK293T cells after 180 s of UV irradiation.^50^

**Table 1:**
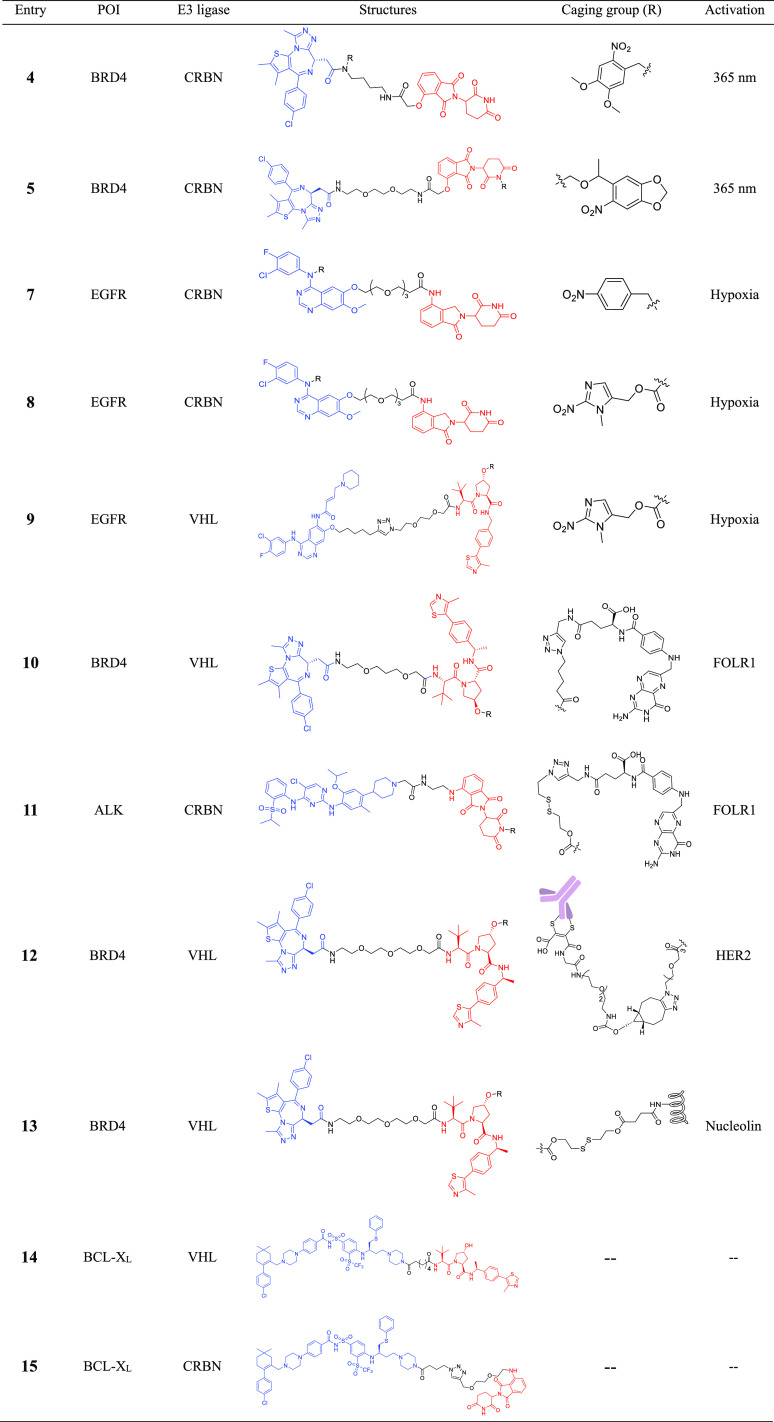
Structures of PROTACs for Increasing Cell or Tissue Selectivity^a^

a Blue: POI ligand, and red: E3 ligand.

#### 3.1.2 Photoswitchable PROTACs

**Figure 4:**
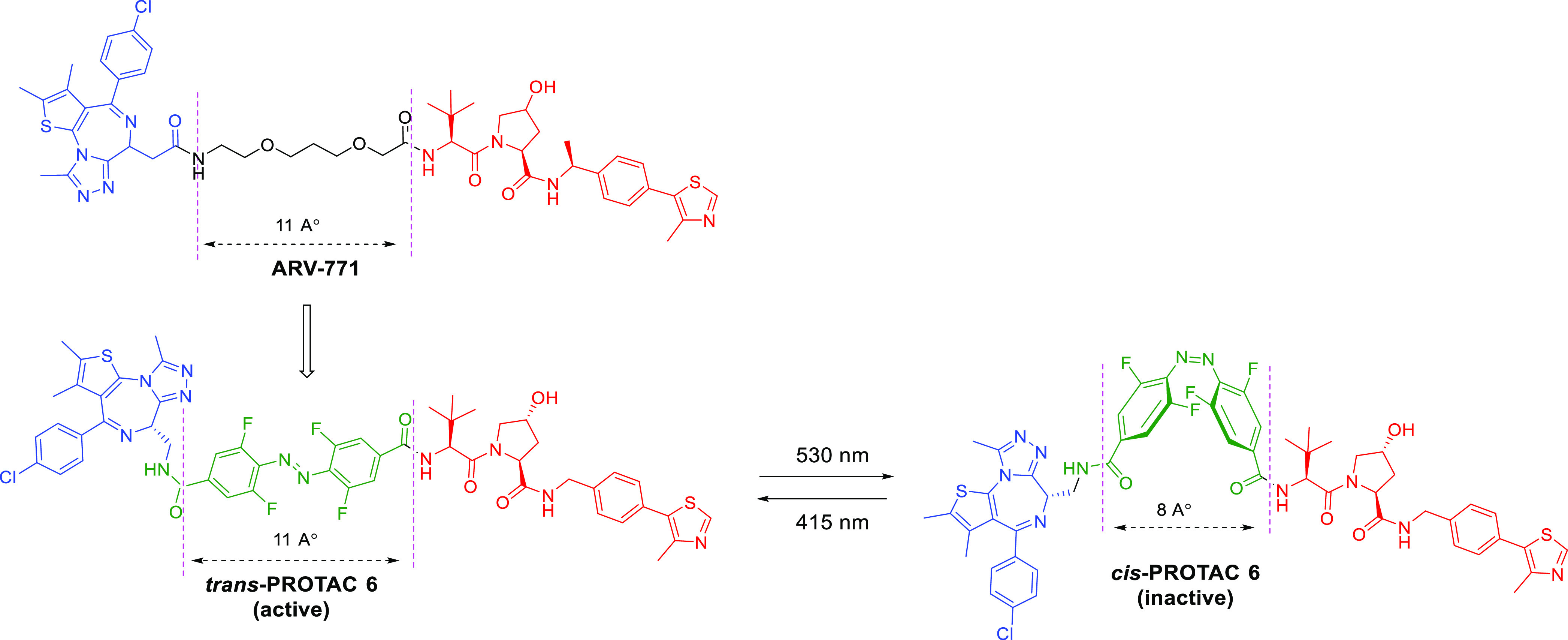
Design strategy of a photoswitchable BET PROTAC. Replacement of the oligoether linker in ARV-771 with *o*-F4-azobenzene generates an isomeric photo-PROTAC pair in which the ***trans***-isomer keeps the maximal distance displayed by ARV-771 while the ***cis***-isomer is considerably shorter.

However, photocaged PROTACs will irreversibly release the active PROTAC molecules upon UV irradiation, which may lead to safety concern.^54^ Photoswitching provides an alternative approach to locally activate PROTACs. Photoswitchable PROTACs were designed by Pfaff et al., who incorporated a bistable *ortho-*tetrafluoroazobenzenes (*o*-F4-azobenzenes) linker between POI ligand and E3 ligase ligand.^54^ They selected ARV-771^55^ as a PROTAC lead structure in which the linker length between POI ligand and E3 ligase ligand is about 11 Å. As shown in Figure 4, replacement of the oligoether linker in ARV-771 with *o*-F4-azobenzene generated an isomeric photo-PROTAC pair in which *trans***-PROTAC 6** (active degrader) maintains an optimal distance of 11 Å between both ligands, while ***cis***-**PROTAC 6** (inactive degrader) has a shorter distance (8 Å). The *trans***-PROTAC 6** could be transformed into *cis***-PROTAC 6** under 530 nm irradiation, generating 68% *cis***-PROTAC 6**, whereas the *cis-***PROTAC 6** could be induced to become *trans***-PROTAC 6** under 415 nm irradiation, generating 95% *trans***-PROTAC 6**. Interestingly, *trans***-PROTAC 6** triggers degradation of BRD2 but not BRD4 in Ramos cells after 18 h, whereas no obvious degradation was observed with *cis***-PROTAC 6**.^54^ In contrast to photocaged PROTACs, the photoswitchable PROTACs offer a reversible on/off switching for targeted protein degradation.

### 3.2 Hypoxia-Activated PROTAC

Hypoxia is a hallmark of most solid tumors and is accompanied by overexpression of proteins including the epidermal growth factor receptor (EGFR).^56,57^ Elevated levels of nitroreductase (NTR) are being observed in the hypoxic region of solid tumors, which enables selective prodrug activation in hypoxic solid tumors.^57^ Similar to photocaged PROTACs, hypoxia-activated PROTACs are designed by attaching a hypoxia-activated leaving group (HALG) to the POI ligand or the E3 ligase ligand. This design enables activation under hypoxic condition by the increased NTR activity, which triggers the release of the active PROTACs, followed by target protein degradation.^58,59^ In 2021, Cheng et al. developed a hypoxia-activated PROTAC (Table 1, entry 7) by introducing the 4-nitrobenzyl group into the EGFR ligand moiety of an EGFR^Del19^-targeting PROTAC.^58^ PROTAC **7** showed sharply decreased binding affinity to EGFR and could achieve selective EGFR^Del19^ degradation in hypoxia instead of normoxia in HCC4006 cells at the concentration of 50 μM with the maximal 87% degradation. Besides the 4-nitrobenzyl group, they also introduced the (1-methyl-2-nitro-1*H*-imidazol-5-yl) methyl group into the EGFR ligand moiety of an EGFR^Del19^-targeting PROTAC. However, the resulting PROTAC **8** (Table 1, entry 8) could still potently bind to EGFR^Del19^, and the discrepancy of the degradation induced by PROTAC **8** between normoxia and hypoxia was not obvious.^58^ In a more recent effort, Shi et al. reported another series of hypoxia-activated PROTAC designed by incorporating the (1-methyl-2-nitro-1*H*-imidazol-5-yl) methyl group on the VHL E3 ligase recruiter.^59^ Among the obtained PROTAC molecules, PROTAC **9** (Table 1, entry 9) could release the active PROTAC molecule under hypoxia, which showed nearly complete deletion of EGFR in HCC-827 cells at the concentration of 250 nM.^59^ Additionally, PROTAC **9** significantly suppressed the tumor growth in a HCC-827 xenograft animal model.^59^ The strategy of hypoxia-activated PROTACs may help to achieve controllable target protein degradation in solid tumors with high level of hypoxia, thus avoiding potential toxicity to normal tissues/cells.

### 3.3 Folate-Caged PROTACs

**Figure 5:**
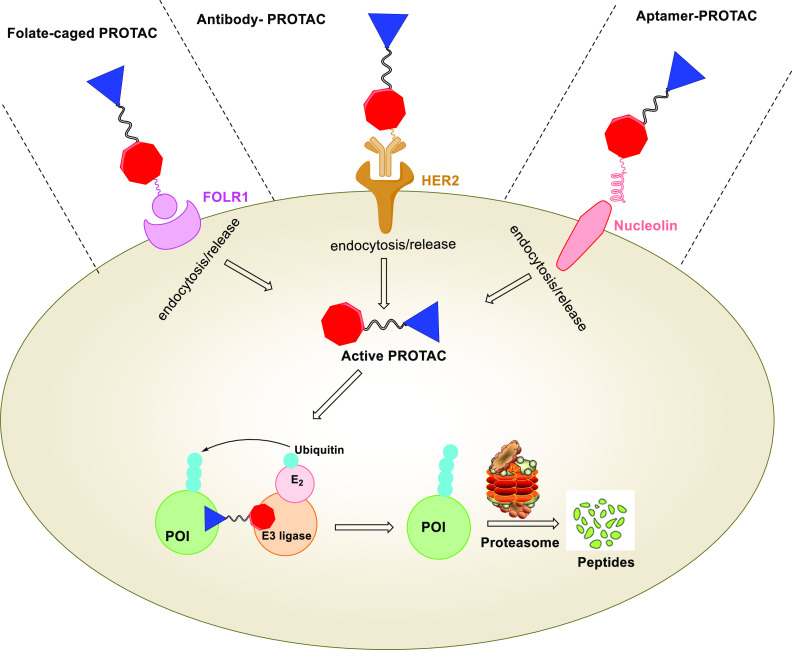
Schematic presentation of the design strategies for folate-caged PROTACs, antibody-PROTAC conjugates (Ab-PROTACs), and aptamer-PROTAC conjugates (APCs). Upon special recognition by the cell membrane receptor (e.g., FOLR1, HER2, and nucleolin), these PROTAC-based conjugates are taken up by cells via endocytosis. Then, the linker is cleaved by hydrolases to release the active PROTAC molecule, leading to target protein degradation.

Folate receptor α (FOLR1) is highly expressed in many human cancers but relatively lowly expressed in normal tissues.^60^ This expression difference provides opportunities for drug targeting by conjugation of folate as a FOLR1 ligand. Conjugation of folate has been utilized as an effective approach for the specific delivery of PROTACs into cancer cells. Folate-caged PROTACs are designed by attaching a folate group on the E3 ligase ligand of PROTACs via a cleavable linker.^61,62^ As shown in Figure 5, upon binding to FOLR1 in the cell membrane, the folate-conjugated PROTACs are transported into cells followed by cleavage of the folate moiety by endogenous hydrolases and reductases. This provides the active PROTAC, which triggers target protein degradation.^61,62^

In 2021, Liu et al. developed a cancer-selective PROTAC on the basis of reported BRD4 PROTAC ARV-771.^61^ The folate-PROTAC **10** (Table 1, entry 10) designed by incorporating folate via an ester bond onto the hydroxyl group of ARV-771, demonstrated comparable BRD4 degradation in cancer cells versus noncancerous normal cells with the parental compound ARV-771. However, the folate-PROTAC failed to deplete BRD4 in both HeLa cells pretreated with free folic acid and HeLa cells without endogenous FOLR1, indicating the FOLR1-depedent BRD4 degradation. In the same year, they reported another folate-caged PROTAC **11** (Table 1, entry 11) designed by attaching a folate group to the pomalidomide moiety of reported ALK PROTAC via a glutathione (GSH)-sensitive linker.^62^ Similar to PROTAC **10**, PROTAC **11** showed FOLR1-dependent NPM-ALK fusion protein degradation. This strategy provides an approach for the targeted delivery of folate-caged PROTACs to FOLR1-expressing cancer cells with the potential to ameliorate toxicity.^62^

### 3.4 Antibody-PROTAC Conjugates (Ab-PROTACs)

Antibody–drug conjugates (ADCs) enable the delivery of a cytotoxic payload specifically to cancer cells. This enables one to achieve a maximal effect on cancer cells whereas undesired effects in noncancer cells can be minimized.^63^ On the basis of the idea of ADCs, the development of antibody-PROTAC conjugates (Ab-PROTAC) have been explored as a new strategy to improve the tissue and cell-type selectivity of PROTACs. Ab-PROTACs are designed by attaching a tumor cell-specific antibody to a PROTAC molecule via a cleavable linker.^64^ Similar to folate-caged PROTACs, the antibody binds selectively to tumor cells, the linker of Ab-PROTACs can be hydrolyzed following antibody–PROTAC internalization, releasing the active PROTAC molecule (Figure 5).^64^ In 2020, Maneiro et al. reported a BRD4-targeting trastuzumab-PROTAC conjugate (Table 1, entry 12).^64^ Trastuzumab is a monoclonal antibody used to treat breast cancers, especially for HER2-positive breast cancers. As expected, Ab-PROTAC **12** could selectively degrade BRD4 only in HER2-positive cells while leaving BRD4 intact in HER2-negative cells. For instance, 100 nM of Ab-PROTAC **12** for 4 h treatment demonstrated almost-complete BRD4 depletion only in HER2-positive cell lines. Significant BRD4 degradation was also observed at the concentration of 50 nM, whereas no degradation was detected at any of the concentrations tested in HER2-negative cell lines. By applying tissue specificity of ADCs to PROTACs, the Ab-PROTACs hold potential to overcome the limitation of PROTAC selectivity.

### 3.5 Aptamer-PROTAC Conjugates (APCs)

Aptamers are single-stranded nucleic acids which can bind to target proteins with high specificity and affinity.^65,66^ Aptamers have been widely used in targeted therapy against human tumors due to small physical size, flexible structure, quick chemical production, versatile chemical modification, high stability and lack of immunogenicity.^66^ In 2021, He et al. developed an aptamer-PROTAC conjugation (APC) approach to improve the tumor-specific targeting ability and *in vivo* antitumor potency of conventional PROTACs.^67^ Upon selective recognition by nucleolin-overexpressing tumor cells, the disulfide bond of the APC is cleaved by GSH followed by the release the active PROTAC molecule (Figure 5). The first aptamer-PROTAC conjugate (APC) was designed by conjugating a BET-targeting PROTAC to nucleolin-targeting aptamer AS1411 (AS) via a GSH-sensitive linker. As expected, the resulting APC **13** (Table 1, entry 13) showed comparable BRD4 degradation with the parental PROTAC (APC **13**, DC_50_ = 22 nM, *D*_max_ > 90% vs parental PROTAC DC_50_ = 13 nM, *D*_max_ > 90%) in highly nucleolin-expressing MCF-7 breast cancer cells.^67^

### 3.6 BCL-X_L_ PROTACs with Low Platelet Toxicity

B-cell lymphoma extra large (BCL-X_L_) is a well-validated drug target in cancer, which is predominantly overexpressed in many solid tumor cells and also in a subset of leukemia cells.^68,69^ However, inhibition of BCL-X_L_ induces on-target and dose-limiting thrombocytopenia, seriously limiting the clinical application of BCL-X_L_ inhibitors.^70−73^ To reduce the platelet toxicity, in 2019, Khan et al. developed a selective navitoclax-based BCL-X_L_ PROTAC degrader by targeting BCL-X_L_ to the VHL E3 ligase, which is minimally expressed in platelets.^72^ The generated PROTAC **14** (Table 1, entry 14) could induce the degradation of BCL-X_L_ in MOLT-4 T-cell acute lymphoblastic leukemia cells with DC_50_ of 63 nM and *D*_max_ of 90.8%. However, minimal reduction (Dmax, 26%) in BCL-X_L_ levels in platelets after incubation with up to 3 μM of PROTAC **14** was observed. In addition, PROTAC **14** was about 4-fold more cytotoxic to MOLT-4 cells than the BCL-2 and BCL-X_L_ dual inhibitor navitoclax and showed almost no effect on the viability of platelets up to a concentration of 3 μM, demonstrating improved antitumor potency and reduced toxicity to platelets compared with navitoclax.^72^ Similar to VHL E3 ligase, CRBN E3 ligase is also poorly expressed in platelets. In 2020, He et al. reported another navitoclax-based BCL-X_L_ PROTAC, which targets BCL-X_L_ to the CRBN E3 ligase for degradation.^73^ The resulting PROTAC **15** (Table 1, entry 15) could induce selective BCL-X_L_ degradation in WI38 nonsenescent cells and WI38 senescent cells but not platelets. Further biological evaluations showed PROTAC **15** could effectively clear WI38 senescent cells and rejuvenate tissue stem and progenitor cells in naturally aged mice without causing severe thrombocytopenia.^73^ These findings demonstrate the potential to use PROTAC technology to reduce on-target drug toxicities by hijacking tissue-specific E3 ligases.

## 4 PROTACs for Recruiting Newly Explored E3 Ligases

To date, there are approximately 600 human E3 ligases; however, only a limited number of them (e.g., CRBN, VHL, MDM2, IAP) have been harnessed to produce effective PROTACs.^6,36,43,74−76^ According to a PROTAC-DB analysis conducted by Jimenez et al., VHL remains the most widely used E3 ligase (36%), and the IAP family can be subdivided into XIAP (14%), cIAP1/BIRC2 (9%), cIAP2/BIRC3 (3%), and unspecified IAPs (5%). In addition, CRBN (14%) and MDM2 (7%) gain the podium of E3 ligase proteins.^36^ However, drug resistance to CRBN and VHL E3 ligase ligand in tumors has already emerged.^44^ In addition, these E3 ligases are generally considered to have low tissue-specific expression. This demonstrates that it is important to explore other E3 ligases that are expressed at a very low level in normal tissues but overexpressed in tumors in order to gain selectivity.^6^ In the past several years, apart from the classically used E3 ligases CRBN, VHL, MDM2, and IAP,^3,43,77^ newly explored E3 ligases including RNF4, RNF114, DCAF16, DCAF15, KEAP1, DCAF11, FEM1B, and L3MBTL3 have also been leveraged for PROTAC design.

### 4.1 RING Finger Protein 114 (RNF114)-Recruiting PROTACs

One of the newly explored E3 ligases is RNF114. This E3 ligase has been addressed in a study by the Nomura’s group in 2019. They found that nimbolide, a terpenoid natural product derived from the Neem tree, could exploited as a recruiter of RNF114.^78^ One of these PROTACs, molecule **16** (Table 2, entry 16), was designed by linking nimbolide to the BET inhibitor JQ1 and enabled RNF114-dependent BRD4 degradation. Although PROTAC **16** treatment in 231MFP cells led to BRD4 degradation after 12 h treatment, PROTAC **16** showed less BRD4 degradation at 1 μM compared to 0.1 and 0.01 μM, which might be due to the hook effect.^78^ While nimbolide could be harnessed to recruit RNF114 for target protein degradation, its high molecular weight, modest chemical stability, and difficulty in synthesis limited its application in PROTAC design. In 2021, they discovered a fully synthetic RNF114 E3 ligase recruiter EN219.^79^ One PROTAC molecule **17** (Table 2, entry 17) obtained by attaching EN219 to BET inhibitor JQ1 with alkyl linker could degrade BRD4 in a RNF114-dependent manner. Similar to the nimbolide-based PROTAC **16**, the EN219-based PROTAC **17** demonstrated robust depletion of BRD4 in 231MFP breast cancer cells with DC_50_ values of 36 and 14 nM for the long and short isoforms of BRD4, respectively. Interestingly, one EN219-based PROTAC **18** (Table 2, entry 18) exhibited preferential degradation of BCR-ABL over c-ABL, compared with several previous BCR-ABL degraders recruiting CRBN or VHL that showed opposite selectivity.^79^

**Table 2:**
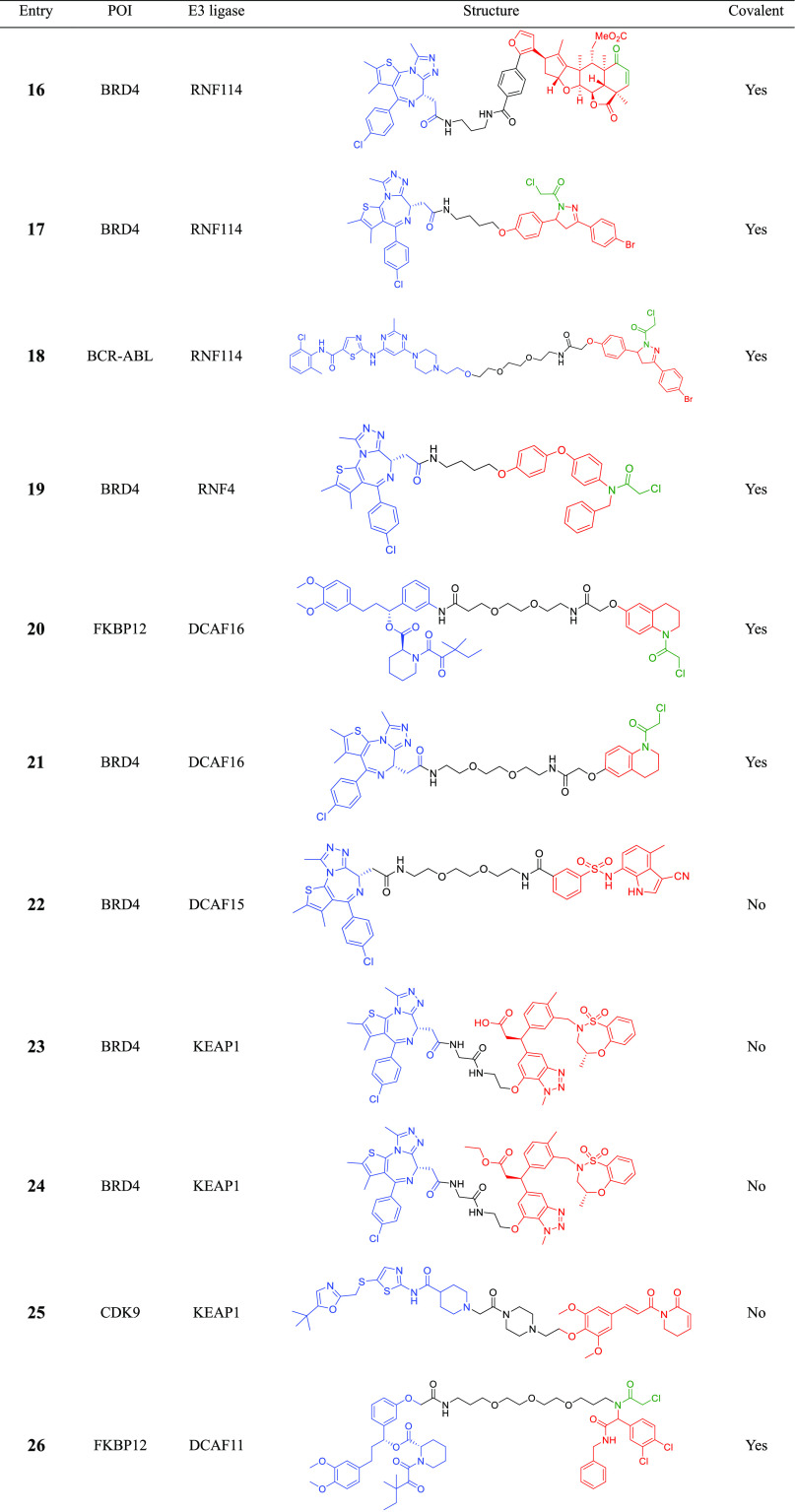
Structures of PROTACs for Recruiting Newly Explored E3 Ligases^a^

a Blue: POI ligand, green: covalent group, and red: E3 ligand.

### 4.2 RING Finger Protein 4 (RNF4)-Recruiting PROTACs

In 2019, Ward et al. identified covalent E3 ubiquitin ligase RNF4 binders via activity-based protein profiling (ABPP)-based covalent ligand screening approaches.^80^ They incorporated this potential RNF4 recruiter into a bifunctional degrader linked to BET inhibitor JQ1. Despite showing lower potency than previously reported JQ1-based degrader MZ1, the RNF4-based PROTAC **19** (Table 2, entry 19) could degrade BRD4 in a time and dose-responsive manner in 231MFP breast cancer cells.^80^

### 4.3 DDB1- and CUL4-Associated Factor 16 (DCAF16)-Recruiting PROTACs

In 2019, Cravatt’s group reported covalent PROTACs containing scout fragments coupled to the FKBP12 ligand SLF.^81^ Among these PROTACs, PROTAC **20** (Table 2, entry 20) could exclusively promote the loss of nuclear FKBP12 without effects on the cytoplasmic localization of FKBP12 under treatment conditions (2 μM, 8 or 24 h) in HEK293T cells. Nevertheless, PROTAC **20** could reduce the nuclear FKBP12 level in WT cells, but not in the DCAF16^–/–^ HEK293T cells, indicating the DCAF16-mediated degradation.^81^ Another BRD4-targeting PROTAC **21** (Table 2, entry 21) was identified using this strategy. PROTAC **21** showed concentration-dependent depletion of BRD4 in HEK293T cells after 24 h treatment. Similarly, PROTAC **21** failed to degrade BRD4 in the DCAF16^–/–^ HEK293T cells, indicating the DCAF16-mediated degradation.^81^

### 4.4 DDB1- and CUL4-Associated Factor 15 (DCAF15)-Recruiting PROTACs

In 2019, DCAF15 was used for PROTAC design by Zoppi et al.^82^ However, the reported DCAF15-based PROTACs did not show obvious target protein degradation. In 2020, Li et al. reported another series of DCAF15-based PROTACs.^83^ Among these obtained compounds, PROTAC **22** (Table 2, entry 22) showed the best BRD4 degradation (DC_50_ = 10.84 ± 0.92 μM, *D*_max_ = 98%) after 16 h treatment in SU-DHL-4 cells. Apart from BRD4, PROTAC **22** could also degrade BRD2 and BRD3 in a dose-dependent manner. Further experiments showed that PROTAC **22** failed to degrade BRD4 in DCAF15 knockout SU-DHL-4 cells, indicating the DCAF15-mediated degradation of BRD4.^83^

### 4.5 Kelch-Like ECH-Associated Protein-1 (KEAP1)-Recruiting PROTACs

KEAP1 functions as a substrate adaptor protein for cullin 3 (CUL3) E3 ligase complexes, leading to polyubiquitinated Nrf2 for proteasomal degradation under normal conditions, which can be exploited as E3 ligase for PROTAC.^84^ Wei et al. reported two KEAP1-based PROTACs containing a selective, noncovalent small-molecule KEAP1 binder and BET inhibitor JQ1.^85^ PROTAC **23** (Table 2, entry 23) featured the KEAP1 ligand with the carboxylic acid group, while PROTAC **24** (Table 2, entry 24) was designed as the prodrug by using the ethyl ester derivative to enhance cell-permeability. Higher concentration (5 μM) of PROTAC **23** significantly depleted BRD4 after 12 h treatment, while lower concentrations (0.5 and 1 μM) of PROTAC **23** failed to show obvious degradation of BRD4, presumably because of its poor cell-permeability. Compared to PROTAC **23**, PROTAC **24** needed longer time to degrade BRD4, with moderate reductions of BRD4 levels (24 h treatment, 0.5 μM), indicating that in-cell hydrolysis of its ethyl ester group is required to release the carboxylic acid group which is crucial for binding to KEAP1.^85^ Piperlongumine (PL) is a natural product that could selectively kill senescent cells in part through induction of OXR1 degradation in a proteasome-dependent manner.^86^ Additionally, PL could be a potential E3 ligase recruiter to generate PROTACs because it could bind to eight different E3 ligases in senescent cells. To validate the hypothesis, Pei et al. described a series of PROTAC molecules consisting of PL and SNS-032 (a selective CDK9 inhibitor).^87^ Among which, compound **25** (Table 2, entry 25) could potently degrade CDK9 at the concentration of 0.1 μM after 6 h treatment. Further biological experiments identified that PROTAC **25** induced CDK9 degradation by the recruitment of the E3 ligase KEAP1.

### 4.6 DDB1- and CUL4-Associated Factor 11 (DCAF11)-Recruiting PROTACs

In 2021, Cravatt’s group described a screening strategy performed with a focused library of candidate electrophilic PROTACs bearing a FKBP12 binder connected to a structurally varied electrophilic group.^88^ Among these covalent PROTACs, PROTAC **26** (Table 2, entry 26) could induce the degradation of both cytosolic and nuclear FKBP12 in DCAF11-WT but not in DCAF11-KO in 22Rv1 cells in a concentration and time-dependent manner, indicating the DCAF11-dependent degradation.^88^ Another PROTAC **27** (Table 2, entry 27) could induce 90% loss of AR at 10 μM after 8 h treatment in 22Rv1 cells. Similarly, PROTAC **27** achieved concentration-dependent degradation of AR in WT cells, not in the DCAF11-KO 22Rv1 cells, indicating the DCAF11-dependent degradation.^88^

### 4.7 Fem-1 Homologue B (FEM1B)-Recruiting PROTACs

Recently, FEM1B was discovered as E3 ligase playing a critical role in the cellular response to reductive stress. Rape et al. found that FEM1B could mediate FNIP1 ubiquitylation and degradation to restore mitochondrial activity, redox homeostasis, and stem cell integrity.^89^ In a more recent effort, Nomura’s group reported a FEM1B-targeting cysteine-reactive covalent ligand, EN106, which can be used as a covalent recruiter for FEM1B in PROTAC.^90^ Two EN106-based PROTACs (Table 2, entry **28** and **29**) stood out as their excellent degradation potency. PROTAC **28** demonstrated the great degradation potency with a DC_50_ of 250 nM and 94% maximal degradation of BRD4 in HEK293T cells.^90^ Additionally, 0.3 μM of PROTAC **29** showed robust degradation of both BCR-ABL and c-ABL after 24 h treatment in K562 cells.^90^

### 4.8 Lethal Malignant Brain Tumor-Like Protein 3 (L3MBTL3)-Recruiting PROTACs

Research showed that the methyl-lysine reader protein, L3MBTL3, could bind to methylated proteins and target them for proteasomal degradation via the Cul4^DCAF5^ complex.^91,92^ On the basis of this natural mechanism, Nalawansha et al. reported several L3MBTL3-recruiting PROTACs.^93^ Among them, PROTAC **30** (Table 2, entry **30**) could recruit L3MBTL3 to indue the FKBP12^F36 V^ degradation in a nuclear-specific manner. Similarly, they also identified another PROTAC **31** (Table 2, entry **31**), which could trigger the BRD2 degradation in multiple cell lines while sparing BRD4 in a dose-dependent manner. Interestingly, these L3MBTL3-recruiting PROTACs show nuclear-specific protein degradation because of the nuclear-restricted expression of L3MBTL3, which can achieve the degradation of nuclear oncogenic proteins with higher selectivity.^93^

## 5 Conclusion and Perspective

In the past two decades, PROTACs have been developed as new therapeutic modalities in drug discovery. Until now, over ten PROTACs are in clinical trials. The event-driven PROTACs hold promise to exploit drug targets that were previously undruggable for the traditional occupancy-driven inhibitors. Nevertheless, the development and application of PROTACs are facing several challenges such as a systemic side effect by PROTAC action outside the diseased area. Furthermore, the design of PROTACs implies often noncompliance with the Lipinski’s rule of five thus increasing the chance for issues with their absorption, metabolism, distribution, and elimination. In the review, we provided a detailed summary of recent strategies to enhance the effectiveness and selectivity of PROTACs. These new PROTAC design strategies can be classified into three main groups: strategies for improving the cellular permeability, strategies for increasing cell or tissue selectivity, and strategies for recruiting newly exploited E3 ligases. However, most PROTACs reported in this review are still in the conceptual stage. Some PROTACs need more biological evaluations, such as *in vivo* studies, safety profiles, and DMPK profiles, the others need to be further optimized to improve their metabolic stability and potency. So, we expect that PROTACs could reach the clinic; however, actually reaching the clinic might be a matter of time. Apart from reaching the clinic the PROTACs are useful tools to probe the biology of proteins in particular with respect to functions for which no small molecule inhibitors can be developed.

To address the PROTACs noncompliance with Lipinski’s rule of five, *in vivo* assembly of a PROTAC was developed using a strategy denoted CLIPTACs. This strategy enables *in vivo* synthesis of a PROTACs from two smaller fragments by click chemistry. Although the two corresponding precursors are much smaller than the preassembled PROTAC molecule, they are still relatively large in size (MW of JQ1-TCO (**1**): 609.19, MW of Tz-Thalidomide (**2**): 571.59) and the physical-chemical properties might be suboptimal. This argues for expansion of the use of other bio-orthogonal reactions apart from tetrazine ligation used in CLIPTACs, such as strain-promoted azide–alkyne cycloaddition (SPAAC).^94^ Although, the CLIPTACs were first described in 2016,^46^ few follow up studies were done for this technology. This might have several reasons. One reason discussed by the authors is that the effectiveness of the CLIPTAC strategy is limited by assembly of the PROTAC molecules before entering the cells. An additional concern is the metabolic stability of the CLIPTAC partner molecules. Alternatively, the CLIPTAC effectiveness might be limited from relatively low reactions yields, which leaves the nonreacted CLIPTAC partners as competing ligands that interfere with formation of the ternary complex needed for PROTAC-mediated protein degradation. Further efforts on the development of CLIPTACs should focus on the discovery of smaller individual precursors, alternative linkage strategies, and precursor activation by intracellular enzymes.

Another weakness of PROTACs is the lack of selectivity for PROTAC-mediated proteolysis in cancer cells versus normal cells. In order to achieve selectivity for cancer cells, several strategies have been developed. These strategies include photochemically controllable PROTACs (PHOTACs), hypoxia-activated PROTACs, folate-caged PROTACs, antibody-PROTAC conjugates (Ab-PROTACs) and aptamer-PROTAC conjugates (APCs), and BCL-X_L_ PROTACs, which proved to be successful as reviewed.^47,48,51^

In the case of hypoxia-activated PROTACs, the PROTACs with a caged POI ligand show lower binding affinity to POI than PROTACs with a caged E3 ligase ligand. Caging of the POI ligand may provide a more pronounced reduction for the on-target toxicity to normal cells. Additionally, it is also important to investigate safety profiles of caging groups. It is worth noting that hypoxia-activated PROTACs are only suitable for solid tumors with a high level of hypoxia. The safety profiles of HALGs also need to be investigated. Folate-caged PROTACs, antibody-PROTAC conjugates (Ab-PROTACs), and aptamer-PROTAC conjugates (APCs) have much larger molecular weight than normal PROTACs, which might lead to poorer oral bioavailability and unfavorable pharmacokinetics. Further attention should be paid to optimizing their DMPK profiles and delivery methods. The folate-caged PROTACs are beneficial for the treatment of cancer types with high expression of FOLR1, and further studies for folate-caged PROTACs are warranted to evaluate their *in vivo* potency and toxicity.

The human genome encodes more than 600 E3 ubiquitin ligases, but only a few of them have been utilized for PROTACs. However, drug resistance to CRBN and VHL E3 ligase ligands in tumors has already emerged. Therefore, there is a need to hijack alternative E3 ligases. Recently, several other E3 ligases have been harnessed successfully for PROTACs. However, most newly explored E3 ligase ligands bind to E3 ligases in a covalent manner and need further optimization to improve potency, selectivity and metabolic stability. Note that most of these new hijackable E3 ligases are ubiquitously expressed in humans. Extensive efforts should also be put in identification of E3 ligases with a tissue or disease-specific expression pattern to improve PROTAC selectivity and increase their therapeutic window.^29^

In addition, PROTACs could pose more challenges with regard to drug metabolism and pharmacokinetics (DMPK) and safety evaluation than traditional small molecules. PROTACs have multiple sites for metabolic degradation thus providing inactivation of the POI ligand or the E3 ligase ligand, thus giving rise to molecules with altered and/or competing activity.^40^ Therefore, the traditional DMPK strategies may not be suitable for PROTAC molecules, which calls for establishment of novel approaches to characterize the pharmacokinetics and metabolite profiling of PROTACs.^40^

Taken together, PROTACs hold great potential for many human diseases. Immense efforts should focus on the development of a next generation of PROTACs with improved selectivity, favorable pharmacokinetics properties, enhanced therapeutic efficacy, and decreased toxicity. PROTACs should not only be developed as potential novel therapeutic agents but also as powerful biological tools to investigate the physiological and pathological functions of enzymes.

## Abbreviations

ABL: tyrosine-protein kinase ABL
Ab-PROTACs: antibody-PROTAC conjugates
APCs: aptamer-PROTAC conjugates
AR: androgen receptor
BCL-X_L_: B-cell lymphoma-extra large
Bcl-2: B-cell lymphoma 2
BCR: breakpoint cluster region protein
BET: bromodomain and extra-terminal domain
BRD: bromodomain-containing protein
CDK: cyclin-dependent kinase
CLIPTAC: in-cell click-formed proteolysis targeting chimeras
CRBN: celeblon
cIAP: cellular inhibitor of apoptosis
DMPK: drug metabolism and pharmacokinetics
DCAF: DDB1- and CUL4-associated factor
ER: estrogen receptor
EGFR: epidermal growth factor receptor
FEM1B: fem-1 homologue B
FKBP: FK506-binding protein
FNIP1: folliculin-interacting protein 1
FOLR1: folate receptor α
HALG: hypoxia-activated leaving group
HBD: hydrogen bond donor
HBA: hydrogen bond acceptor
HEK 293 cells: human embryonic kidney 293 cells
HER2: human epidermal growth factor receptor 2
KEAP1: kelch-like ECH-associated protein 1
L3MBTL3: lethal (3) malignant brain tumor-like protein 3
MW: molecular weight
MDM2: mouse double minute 2 homologue
NTR: nitroreductase
Nrf2: nuclear factor erythroid 2-related factor 2
POI: protein of interest
PHOTACs: photochemically controllable PROTACs
PL: piperlongumine
PROTACs: proteolysis targeting chimeras
PSA: polar surface area
RINF: ring finger protein
SPAAC: strain-promoted azide–alkyne cycloaddition
TPD: targeted protein degradation
TCO: *trans*-cyclo-octene
UPS: ubiquitin-proteosome system
VHL: von Hippel–Lindau.
